# A Genetic Polymorphism in TOX3 Is Associated with Survival of Gastric Cancer in a Chinese Population

**DOI:** 10.1371/journal.pone.0072186

**Published:** 2013-09-17

**Authors:** Xiaojing Zhang, Haixia Zhu, Xiaomin Wu, Meilin Wang, Dongying Gu, Weida Gong, Zhi Xu, Yongfei Tan, Yongling Gong, Jianwei Zhou, Cuiju Tang, Na Tong, Jinfei Chen, Zhengdong Zhang

**Affiliations:** 1 Department of Oncology, Nanjing First Hospital, Nanjing Medical University, Nanjing, P. R. China; 2 Core Laboratory, Nantong Cancer Hospital, Nantong, China; 3 Department of Molecular & Genetic Toxicology, School of Public Health, Nanjing Medical University, Nanjing, China; 4 Department of Surgery, Yixing Cancer Hospital, Yixing, China; 5 Department of Molecular Cell Biology & Toxicology, School of Public Health, Nanjing Medical University, Nanjing, China; 6 Department of Gynecologic Oncology, Zhejiang Cancer Hospital, Hangzhou, Zhejiang, P.R. China; 7 Department of Oncology, Yancheng No.1 People's Hospital, Yancheng, P.R. China; MOE Key Laboratory of Environment and Health, School of Public Health, Tongji Medical College, Huazhong University of Science and Technology, China

## Abstract

**Purpose:**

Recently, genetic polymorphism (rs3803662C>T) in *TOX3* was reported to induce the risk of breast cancer. In this study, we hypothesized that rs3803662 could influence gastric cancer survival outcomes.

**Methods:**

With multiplex SNaPshot method, we genotyped *TOX3* rs3803662 in 880 gastric patients with surgical resection. The association between genotype and survival outcomes was performed by the Kaplan-Meier method, Cox regression analysis models and the log-rank test.

**Results:**

There was no association in the analyses of rs3803662 and survival of gastric cancer. However, the stratified analysis by histology showed that rs3803662 CT/TT genotype was associated with a significantly better survival for diffuse-type gastric cancer (log-rank *p* = 0.030, hazard ratio [HR]  = 0.67, 95% confidence interval [CI]  = 0.46–0.96), than the CC genotype. In addition, this favorable effect was especially obvious among gastric cancer patients with tumor size >5 cm, T3 and T4 depth of invasion, lymph node metastasis, no drinking, no distant metastasis, no chemotherapy and gastric cardia cancer.

**Conclusions:**

*TOX3* rs3803662 might play an important role in the prognostic outcome and treatment of gastric cancer, especially perhaps further help in explaining the reduced risk of death associated with diffuse-type gastric cancer.

## Introduction

Gastric cancer is the fourth common type of malignant tumor in the world and the leading cause of cancer deaths in China, Japan and Eastern European countries [1]. A systematic treatment including surgery, chemotherapy, radiotherapy and target therapy may provide a cure for patients with advanced gastric cancer [2]. Despite this, survival in patients with advanced gastric cancer remains poor. Gastric cancer is a complex and multifactor disease that is thought to result from an interaction between genetic background and environmental factors. Some studies have suggested that *Helicobacter pylori* (*H. pylorus*) infection, salt-preserved food consumption and tobacco smoking are major exogenous factors and increase the risk of gastric cancer [3], [4]. Extensive epidemiological studies have demonstrated that genetic variants, particularly single nucleotide polymorphisms (SNPs), are likely to modulate the effect of environmental risk factors through modifying functions of various biological pathways involved in gastric carcinogenesis in response to environmental exposure. In recent years, several common low-penetrant genes have been identified as potential gastric cancer susceptibility genetic variants, such as Glutathione S-transferase M1 (*GSTM1*) polymorphism. It has been found genetic variant may be a useful marker for predicting the prognosis of patients with gastric cancer. Previous studies reported *PSCA* rs2294008 and *APE1* rs1760944 are associated with gastric cancer survival [5], [6].

*TOX3* gene, a member of the high mobility group family of non-histone chromatin proteins, also termed trinucleotide repeat containing 9 (*TNRC9*) gene is located at chromosome 16q12 [7]. This gene regulates Ca2+-dependent neuronal transcription through interaction with the cAMP-response-element-binding protein (CREB) [8]. In normal human tissues, *TOX3* is largely expressed in the central nervous system (CNS), in the ileum, and within the brain in the frontal and occipital lobe. *TOX3* overexpression induces transcription involving isolated estrogen-responsive elements and estrogen-responsive promoters, and protects neuronal cells from cell death caused by endoplasmic reticulum stress or BAX overexpression through the induction of anti-apoptotic transcripts and repression of pro-apoptotic transcripts [9]. It has been suggested that rs3803662 (a C>T transition) in *TOX3* was associated with an increased risk of breast cancer in both BRCA1 and BRCA2-mutation carriers and estrogen receptor (ER) positive patients [10]. Additionally, Fasching et al. reported rs3803662 (*TOX3*) was associated with OS of breast cancer [11]. This association was seen similarly in other study which showed that when survival was analysed according to molecular subtypes, patients diagnosed with luminal A tumours who carried the risk allele had shorter OS as compared to patients homozygous for the non-risk allele [12].

However, GWAS focused on a loci polymorphism at *TOX3* gene that is associated with breast cancer survival, especially in estrogen receptor (ER) positive patients. Some studies suggested that ER expression was associated with survival of gastric cancer [13]. Therefore, the variant identified in breast cancer studies may have the same impact on gastric cancer risk. An association of genetic variation in *TOX3* with gastric cancer survival was conducted in our study. We hypothesize that *TOX3* rs3803662 is associated with gastric cancer survival in a Chinese population, which can be identified as an independent prognostic marker of gastric cancer survival.

### Study population

The study was conducted on a total of 1022 gastric cancer patients with surgical resection of the tumor at Yixing People's hospital, Yixing City, the People's Republic of China (P.R. China), from January 1999 to December 2006 [6]. All patients were neither administrated by adjuvant radiotherapy nor chemotherapy before surgical resection. In our study population, all analyses were restricted to Chinese. Within a maximum of 119.0 months follow-up period, 78 patients were excluded from our study for lacking of enough follow-up information. The present study was approved by the Institutional Ethics Review Board of Nanjing medical university, and all participants gave written informed consent.

### Outcomes collection

Overall survival was the main study endpoint. Patients alive on the last follow-up date were considered censored and gastric cancer -related deaths were defined as deaths. The survival time was defined as the date from cancer surgery to gastric cancer -related deaths or the last follow-up. Date of death was obtained from inpatient and outpatient records or the relatives of patients by follow-up telephone calls. Information of pathologic parameters on tumor site, histotype, invasion, lymph node, distant metastasis status, drinking and smoking status were also obtained for gastric cancer patients. Lauren's criterion was used in classifying the tumors into intestinal-type or diffuse-type gastric cancer [14]. TNM stage of the disease was measured in accordance with American Joint Commission for Cancer Staging (AJCCS) [15]. There were 306 patients treated with adjuvant chemotherapy with different regimens after surgery. The therapeutic regimen included FOLFOX4, FOLFOX6, XELOX and so on. Our study was approved by the Institutional Review Board of Nanjing Medical University, Nanjing, P. R. China.

## Patients and Methods

### Genotyping

Genomic DNA was extracted from tumor specimens by proteinase K digestion, isopropanol extraction and ethanol precipitation [6]. The *TOX3* (rs3803662) SNPs were examined by multiplex SNaPshot technology using an ABI fluorescence-based assay allelic discrimination method (Applied Biosystems, Foster city, CA, USA) as described previously [16]. The SNPs were analyzed by using ABI3130 genetic analyzer and the genotypes were determined by using Genemapper4.0 software (Applied Biosystems). In fact, 880 eligible gastric patients were enrolled in the final analysis, 64 cases were excluded from further analyses because of DNA quality failing in genotyping. About 10% of the samples were selected at random for confirmation by repeated genotyping; the results were 100% concordant.

### Statistical analysis

The influence of *TOX3* variant on gastric cancer survival was estimated by the Kaplan-Meier survival curves and the log–rank test. Three genetic models (codominant, dominant and recessive) were used to assess the association of *TOX3* rs3803662 with survival outcome of gastric cancer patients. All *P* values in this study were 2-sided. *P*<0.05 was considered statistically significance. The crude hazard ratios (HRs), their 95% confidence intervals (CIs) and adjusted HRs were estimated by Cox regression analysis. Cox stepwise regression analysis was also performed to determine prognostic factor in gastric cancer, with a significance level of *P*<0.05 for entering and *P*>0.10 for removing the respective explanatory variables. Mean survival time was provided when the median survival time (MST) could not be calculated. All the statistical analyses were performed with SPSS software.

## Results

### The characteristics and clinicopathological features of the patients

The characteristics and clinicopathological features of the final 880 gastric cancer patients in this study are summarized in Table 1. The percentage of males was 76.9%. The number of patients died during follow-up was 408. Univariate Cox regression analysis of the pathologic parameters revealed that tumor size, histology, depth of invasion, lymph node metastasis, distant metastasis and TNM stage were significantly associated with survival time (all *P*<0.05, log-rank test). Specifically, diffuse-type gastric cancer patients had a significantly higher risk of death, compared with intestinal-type gastric cancer patients (HR  = 1.46, 95% CI  = 1.19–1.79). And patients with tumor size ≤5 cm had a 41% significantly increased survival, compared to those with tumor size>5 cm (HR  = 1.41, 95% CI  = 1.16–1.71). In addition, as the depth of invasion and TNM stage increased, the lymph node metastasis and distant metastasis expanded, and then the survival time of gastric cancer reduced significantly accordingly (log–rank *P*<0.001 for depth of invasion, TNM stage and the lymph node metastasis; log–rank *P* = 0.031 for distant metastasis).

**Table 1:** Patients' characteristics and clinical features.

Variable	Patients, n = 880 (%)	Deaths, n = 408	MST (months)	Log-rank *p*	HR (95% CI)
**Age (years)**					
≤60	413 (46.9)	189	97.0	0.353	1.00
>60	467 (53.1)	219	60.0		1.10(0.90–1.33)
**Sex**					
Male	677 (76.9)	310	70.0	0.331	1.00
Female	203 (23.1)	98	63.0		1.12(0.89–1.40)
**Tumor size**					
≤5cm	546 (62.1)	231	73.5^3^	0.001	1.00
>5cm	334 (37.9)	177	48.0		1.41(1.16–1.71)
**Histological types** 1					
Intestinal	371 (42.2)	143	76.9^3^	<0.001	1.00
Diffuse	505 (57.4)	262	50.0		1.46(1.19–1.79)
others	4 (0.004)	3	11.0		2.72(0.87,8.53)
**Tumor site**					
Non-cardia	584 (66.4)	276	67.0	0.277	1.00
cardia	296 (33.6)	132	78.0		0.89(0.73–1.10)
**Depth of invasion^2^**					
T1	134 (15.2)	43	85.2^3^	<0.001	1.00
T2	186 (21.1)	73	73.1^3^		1.34(0.92–1.95)
T3	520 (59.1)	271	48.0		1.98(1.43–2.74)
T4	38 (0.04)	21	29.0		2.20(1.31–3.71)
**Lymph node metastasis**					
N0	348 (39.5)	121	81.6^3^	<0.001	1.00
N1/N2/N3	532 (60.5)	287	42.0		1.84(1.49–2.28)
**Distant metastasis**					
M0	829 (94.2)	379	74.0	0.031	1.00
M1	51 (5.8)	29	27.0		1.51(1.03–2.20)
TNM stage					
I	240 (27.3)	79	84.0^3^	<0.001	1.00
II	174 (19.8)	70	68.7^3^		1.32(0.96–1.83)
III	379 (43.1)	212	38.0		2.11(1.63–2.74)
IV	87 (0.08)	47	47.0		1.98(1.38–2.85)
**Drinking status**					
NO	825 (93.8)	379	70.0	0.346	1.00
YES	55 (6.2)	29	39.0		1.20(0.82–1.75)
**Chemotherapy**					
NO	587(66.7)	276	70.0	0.656	1.00
YES	293(33.3)	132	62.0		1.05(0.85–1.29)
**Smoking status**					
NO	808 (91.8)	376	67.0	0.699	1.00
YES	72 (8.2)	32	97.0		0.93(0.65–1.34)

1 Exclude four patients with gastrointestinal stromal tumor and lymphoma.^ 2^Two cases were not available for the information. ^3^Mean survival time was provided when MST could not be calculated.

### Effects of rs3803662 on gastric cancer survival

**Figure 1:**
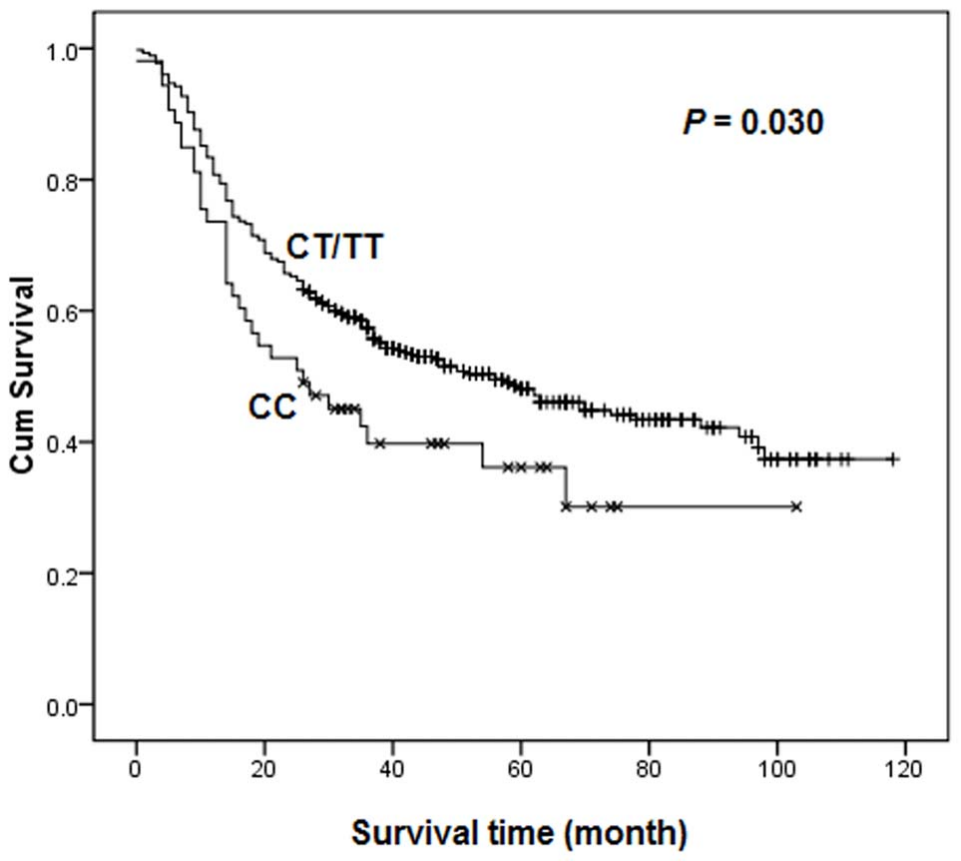
Overall survival of *TOX3* rs3803662 dominant genotypes in diffuse-type gastric cancer patients.

**Figure 2:**
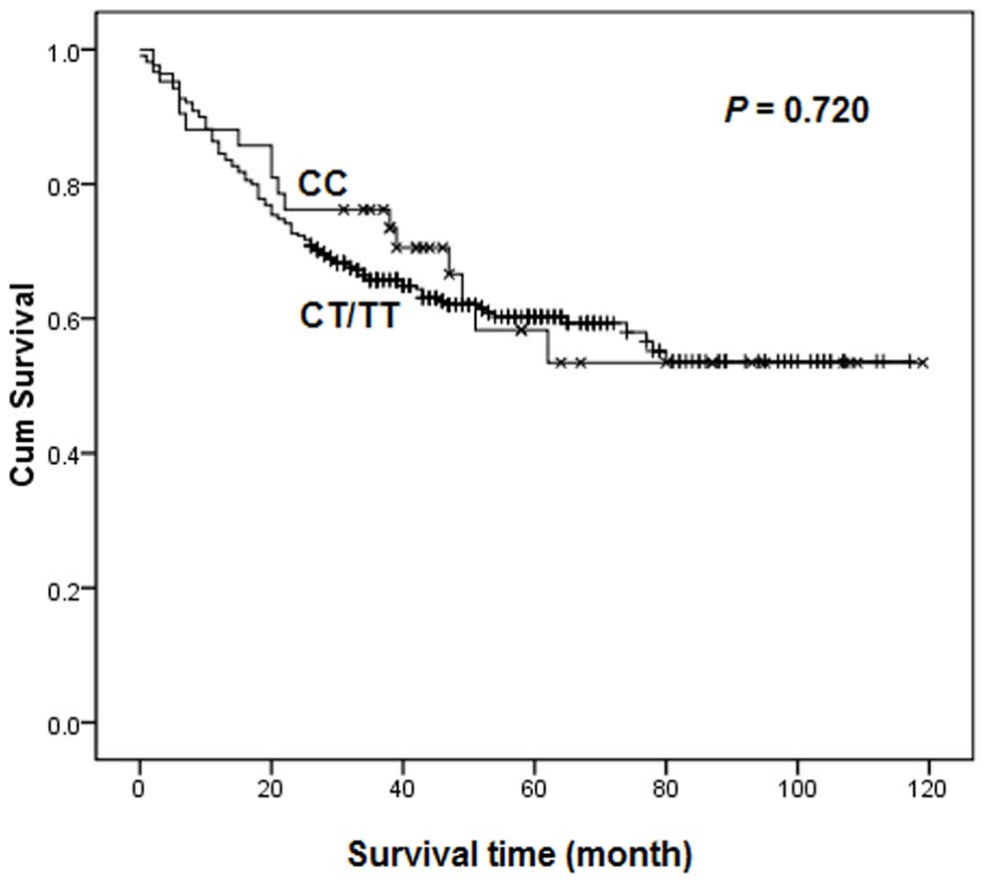
Overall survival of *TOX3* rs3803662 dominant genotypes in intestinal-type gastric cancer patient.

As shown in Table 2, the associations of *TOX3* rs3803662 genotypes with gastric cancer survival in different genetic models were assessed by Cox regression analyses. No statistically significant differences were found between the survival of gastric cancer and genotypes in any genetic models. To further evaluate the association, the patients were stratified by histology with intestinal-type and diffuse-type gastric cancer. As a result, rs3803662 was found to be associated with a significantly favorable effect among diffuse-type (log-rank *P* = 0.030, Fig. 1), but not intestinal-type (log-rank *P* = 0.720, Fig. 2) gastric cancer patients in dominant model. Cox regression analyses revealed that patients with diffuse-type gastric cancer carrying the CT/TT genotype reduced a 33% risk of death significantly (HR  = 0.67, 95% CI  = 0.46–0.96), comparing to those who were CC carriers.

**Table 2:** Association between TOX3 rs3803662 polymorphism and gastric cancer patients' survival.

Genetic models	Genotypes		All cases			
		Patients, n = 880	Deaths, n = 408	MST (months)	Log-rank *p*	HR (95% CI)^1^
Codominant model	CC	96	50	49.0	0.194	1
	CT	522	230	80.0		0.77(0.57–1.05)
	TT	262	128	60.0		0.89(0.64–1.23)
Dominant model	CC	96	50	49.0	0.200	1
	CT/TT	784	358	74.0		0.81(0.60–1.09)
Recessive model	CC/CT	618	280	74.0	0.332	1
	TT	262	128	60.0		1.10(0.89–1.36)
				Intestinal-type cases		
Genetic models	Genotypes	Patients, n = 371	Deaths, n = 143	MST (months)	Log-rank *p*	HR (95% CI)
Codominant model	CC	42	16	77.7^2^	0.308	1
	CT	217	78	78.5^2^		0.95(0.55–1.64)
	TT	112	49	78.0		1.28(0.73–2.27)
Dominant model	CC	42	16	77.7^2^	0.720	1
	CT/TT	329	127	75.6^2^		1.06(0.63–1.79)
Recessive model	CC/CT	259	94	79.4^2^	0.125	1
	TT	112	49	78.0		1.34(0.94–1.89)
				Diffuse-type cases		
Genetic models	Genotypes	Patients, n = 505	Deaths, n = 262	MST (months)	Log-rank *p*	HR (95% CI)
Codominant model	CC	53	33	26.0	0.078	1
	CT	303	150	56.0		0.65(0.44–0.95)
	TT	149	79	48.0		0.70(0.47–1.06)
Dominant model	CC	53	33	26.0	0.030	1
	CT/TT	452	229	56.0		0.67(0.46–0.96)
Recessive model	CC/CT	356	183	50.0	0.869	1
	TT	149	79	48.0		1.02(0.78–1.33)

1 Adjusted for age, sex. ^2^Mean survival time was provided when MST could not be calculated. CI, confidence interval; HR, hazard ratio.

### Stratified analyses among diffuse-type gastric cancer

*TOX3* rs3803662 was investigated with survival of diffuse-type gastric cancer patients by stratified analysis of tumor size, histology, depth of invasion, lymph node metastasis, distant metastasis, TNM stage, drinking status, and tumor site. As shown in Table 3, after adjusting for covariates including age and sex, the reduced risk of death was much evident among patients with tumor size >5 cm (HR  = 0.35, 95% CI  = 0.21–0.56), T3 and T4 depth of invasion (HR  = 0.63, 95% CI  = .42–0.94 for T3; HR  = 0.07, 95% CI  = 0.01–0.83 for T4), lymph node metastasis (HR  = 0.62, 95% CI  = 0.41–0.93), no distant metastasis (HR  = 0.68, 95% CI  = 0.46–1.00), no drinking (HR  = 0.63, 95% CI  = 0.43–0.92), no chemotherapy (HR  = 0.54, 95% CI  = 0.34–0.86) and gastric cardiac cancer (HR  = 0.50, 95% CI  = 0.25–1.00). Although patients with T1 depth of invasion and stage I showed a similar better outcome, there might have been a suboptimal statistical power to detect a statistical difference in the survival time because of small number of patients in these subgroups. In contrast, there seemed to be a poor survival for patients with tumor size ≤5 cm and drinking although not statistically significant. In addition, a multivariate stepwise Cox regression analysis was also used to confirm an independent role of rs3803662 genotypes in diffuse-type gastric cancer survival. Five variables (drink, lymph node metastasis, tumor site, chemotherapy and rs3803662) were included in the regression model with a significance level of 0.05 for entering and 0.10 for removing a variable (data not shown). When age and sex were included in the final model, the rs3803662 CT/TT genotype was showed to be a significantly favorable predictor for survival of diffuse-type gastric cancer. (CT /TT vs. CC: HR  = 0.63, 95% CI  = 0.43–0.90) (Table 4).

**Table 3:** Stratified analysis of TOX3 rs3803662 genotypes associated with diffuse-type gastric cancer patients' survival.

Variable	Genotypes (death/patients)		
	CC	CT/TT	HR (95% CI)^1^
Total	33/53	229/452	0.67(0.46–0.96)
**Tumor size**			
≤5cm	12/30	120/252	1.19(0.66–2.15)
>5cm	21/23	109/200	0.35(0.21–0.56)
**Depth of invasion**			
T1	0/2	9/20	0.00
T2	3/7	31/80	0.85(0.26–2.80)
T3	29/43	173/323	0.63(0.42–0.94)
T4	1/1	16/29	0.07(0.01–0.83)
**Lymph node metastasis**			
N0	6/13	40/111	0.79(0.33–1.87)
N1/N2/N3	27/40	189/341	0.62(0.41–0.93)
**Distant metastasis**			
M0	29/48	214/425	0.68(0.46–1.00)
M1	4/5	15/27	0.69(0.22–2.20)
**TNM stage**			
I	0/4	19/56	0.00
II	8/13	33/84	0.52(0.24–1.12)
III	19/29	149/259	0.69(0.43–1.12)
IV	6/7	28/53	0.44(0.18–1.13)
**Drinking status**			
NO	32/51	209/426	0.63(0.43–0.92)
YES	1/2	20/26	2.48(0.30–20.70)
**Chemotherapy**			
NO	21/28	153/280	0.54(0.34–0.86)
YES	12/25	76/172	0.84(0.45–1.56)
**Tumor site**			
Non-cardia	23/36	171/323	0.74(0.48–1.14)
cardia	10/17	58/129	0.50(0.25–1.00)

1 Adjusted for age, sex.

**Table 4:** Stepwise Cox regression analysis on diffuse-type gastric cancer patients' survival.

Final variables	β	SE	HR	95% CI	*P*
Age	0.093	0.126	1.10	0.86–1.40	0.460
Sex (female vs. male)	−0.023	0.147	0.98	0.73–1.30	0.870
Drink (no vs. yes)	0.606	0.238	1.83	1.15–2.92	0.010
Lymph node metastasis (N1/N2/N3 vs. N0)	0.659	0.164	1.93	1.40–2.67	<0.001
Tumor site(non-cardia vs. cardia)	−0.374	0.146	0.69	0.52–0.92	0.010
Chemotherapy (no vs. yes)	−0.269	0.134	0.76	0.59–0.99	0.045
rs3803662 (CT/TT vs. CC)	−0.469	0.187	0.63	0.43–0.90	0.012

## Discussion

In our present study, we investigated the effect of *TOX3* rs3803662 on survival of gastric cancer patients. Dominant model was the best-fitting model for *TOX3* rs3803662. The rs3803662 CT/TT genotype exhibited a significant association with better survival among diffuse-type gastric cancer patients. Such a difference was not found for intestinal-type gastric cancer patients. The mechanism of gastric cancer survival patterns which were varied by histological subtypes remains to be elucidated. The results differing in the two major histological types of gastric cancer may be due to their diverge in many clinical characteristics and molecular, including their etiology, epidemiology, carcinogenesis and progression, mRNA and/or protein expression profile, gene copy numbers, microsatellite instability, loss of heterozygosity and mutation profile [17]. Furthermore, given the effects of *TOX3* on neuronal cells [9], rs3803662 increasing the survival of diffuse-type gastric cancer in a dominant model might be attribute to inhabit a host cell into a tumor cell or cancer cells multiply rapidly by unknown mechanisms. In our study, no drinking is a protective factor for diffuse-type gastric cancer patients' survival, it can be seen that primary prevention popularized into people's daily life will be beneficial to decrease the risk of death among diffuse-type gastric cancer. To the best of our knowledge, this is the first study to examine the association between *TOX3* rs3803662 and gastric cancer survival. Our findings provide support for the genome-based studies of gastric cancer survival, especially to Chinese populations.

Gene-gene interaction is a hot topic in genetic epidemiology, including prognosis study. Not a single locus can fully explain their genetic susceptibility and prognosis. Liu et al. reported that the multifactor interactions among polymorphisms in MMP-2, FASL and FAS play more important role in the development of GCA. Similarly, they also found that a gene-dose effect in association with prognosis of NSCLC patients [18], [19]. Winkelmann et al. observed the rs3803662 which is in low LD with rs3104767 showed association to RLS (λ-corrected nominal P_GWA_  = 7.29×10^−7^). However, logistic regression analysis conditioned on rs3104767 demonstrated that this association is dependent on rs3104767 [20]. Therefore the results above raised the possibility that a combination of SNPs within *TOX3* or elsewhere in the gene act cumulatively to increase the risk.

Riaz et al. found that the risk alleles of rs3803662 near the *TOX3* gene was associated with a lower expression of *TOX3* mRNA in breast cancer and hypothesized a tumor suppressor role of this gene [21]. These findings suggest the critical roles of *TOX3* in diseases. It is noted that the specific SNPs and genes related to prognosis may differ from those involved in susceptibility. Nevertheless, the variant identified in breast cancer studies may have the same impact on gastric cancer risk. Thus, whether rs3803662 which changed in the prognosis of gastric cancer may also influence high risk for the disease in patients is needed to warrant. If validated in further research, *TOX3* rs3803662 might also be taken as potential genetic marker for gastric cancer susceptibility except for prognosis.

Some limitations should be considered when we interpret the results. First, our study had been implemented in populations of Chinese; whether there is some difference for the same SNP in the characteristics and survival of gastric cancer among different populations needs to be further tested. Second, more research using larger patient populations is needed to confirm our findings due to a relatively small sample size in stratified analysis as well as to exclude the possibility of association occurred by chance. Third, *H. pylorus* is generally accepted to be the major etiologic agent for peptic ulcer disease as well as for gastric adenocarcinoma [22]. However, we did not investigate on H. pylori infection because there was not enough patient clinical information.

In conclusion, *TOX3* rs3803662 plays an important role in the survival of gastric cancer patients, especially can be considered as an independent prognostic marker of diffuse-type gastric cancer. Importantly, our results offer a very promising prospect for exploring and translating our findings into further potential clinical and diagnostic therapeutic applications in gastric cancer.
